# The Role of Aberrant Metabolism in Cancer: Insights Into the Interplay Between Cell Metabolic Reprogramming, Metabolic Syndrome, and Cancer

**DOI:** 10.3389/fonc.2020.00942

**Published:** 2020-06-11

**Authors:** Yina Yu, Liang Gong, Jun Ye

**Affiliations:** ^1^Department of Gastroenterology, The Second Affiliated Hospital, Zhejiang University School of Medicine, Hangzhou, China; ^2^Department of Otolaryngology, Cixi People's Hospital, Ningbo, China

**Keywords:** metabolic syndrome, metabolic reprogramming, cancer, metastasis, metabolism-targeting therapy

## Abstract

Metabolic syndrome (MetS) is characterized by hyperglycemia, hypertension, dyslipidemia and abdominal obesity. Patients with MetS or other metabolic disorders are more susceptible to cancer development and recurrence and have a worse long-term prognosis. Moreover, the metabolic reprogramming observed in cancer cells has also been described as one of the new hallmarks of cancer. Thus, aberrant metabolism has been proposed as an important risk factor for cancer. Chronic inflammation, reactive oxygen species (ROS), and oncogenic signaling pathways are considered as main potential triggers. Considering the strong association between metabolism and cancer, metabolism-modulating drugs, including metformin and statins, as well as adopting a healthy lifestyle, have been extensively investigated as strategies to combat cancer. Furthermore, strategies that interfere with the metabolic rewiring of cells may also have potent anti-cancer effects. In this article, we provide a comprehensive review of current knowledge on the relationship between aberrant metabolism and cancer and discuss the potential use of metabolism-targeting strategy for the treatment of cancer.

## Introduction

Metabolic Syndrome (MetS) is a constellation of metabolic risk factors and a significant cause of morbidity and mortality. The five main components of MetS are abdominal obesity, hyperglycemia, high blood pressure, hypertriglyceridemia, and low high-density lipoprotein (HDL)-cholesterol levels (1, 2). Although the reported prevalence of MetS varies among different studies, its prevalence is increasing at an alarming rate worldwide.In Western countries, one in five adults are diagnosed with MetS (3). Cancer is the leading cause of death and, despite advances in cancer prevention, its incidence remains exceptionally high. Given the high prevalence of metabolic disorders and cancer, as well as the fact that many patients suffer from both, the potential association between MetS and cancer has been extensively investigated.

Several studies have suggested that people who suffer from MetS have higher chances of developing cancer; the rate of cancer recurrence and mortality are also higher. A recent study has shown that people diagnosed with MetS have a 33% higher cancer mortality rate compared to patients who have no metabolic disorders (4). Furthermore, the number of MetS components was directly proportional to the cancer-related mortality rate (4). Among the MetS components, obesity and hyperglycemia have been suggested as the determinants of tumor-associated clinicopathology. Furthermore, mounting evidence highlights the impact of certain metabolic disorders on the risk for several types of cancer, including colorectal, prostate, pancreatic, renal, liver, post-menopausal breast, and endometrial cancer (5–8). Moreover, malignant cells acquire changes in anabolic and catabolic pathways to meet their high metabolic and energy demands, a phenomenon known as metabolic reprogramming (9). Importantly, metabolic reprogramming has emerged as a hallmark of cancer and has been shown to be involved in cancer initiation, progression, and metastasis, as well as the survival of cancer cells and the development of resistance to antitumor therapies.

The identification of metabolic reprogramming and metabolic syndrome as important regulators of cancer development and progression has provided a rationale for the development of metabolism-targeting therapies as a promising therapeutic approach for cancer. Herein, we provide a comprehensive review of the current understanding related to the association between metabolism and cancer, the potential underlying mechanisms, and emerging metabolism-targeting anticancer therapies.

## Cancer Cell Metabolic Reprogramming

### Rewiring of Cancer Cell Metabolism

The rapidly dividing cancer cells have high demands for energy and nutrients to meet their high metabolic needs. Consequently, the cellular metabolism undergoes a rewiring during malignant transformation. Importantly, cancer cells switch from oxidative phosphorylation to aerobic glycolysis to generate ATP (adenosine triphosphate), a phenomenon commonly referred to as the Warburg effect (10). Several mechanisms have been identified to mediate the metabolic rewiring of cancer cells. Genome instability play a vital part in the alternation of energy metabolism (11). The aberrant activation of certain oncogenes such as K-ras (12), MYC (13), mTOR (14), and P53 (15), have been identified as cell-autonomous mechanisms regulating various aspects of the Warburg effect. Somatic mutations in the mitochondrial genome (mtDNA) or changes in the mtDNA content leading to mitochondrial dysfunction have been associated with an increased glycolytic rate in malignant cells (16).

Apart from cell-autonomous mechanisms, extrinsic factors have also been identified as driving metabolic reprogramming in cancer cells. The tumor microenvironment is often hypoxic in solid cancers, leading to the activation of HIF-1α. The transcriptional activity of HIF-1α inhibits mitochondrial respiratory chains and induces glycolysis, among other things. Several types of stromal cells, including tumor-associated macrophages (TAM), have also been implicated in establishing a hypoxic tumor microenvironment and promoting aerobic glycolysis (17), subsequently leading to metabolic reprogramming in cancer cells (18).

Increased uptake of glutamine and enhanced glutaminolysis have been identified as a hallmark of cancer cells. Glutamine is essential in cancer cells and has been implicated in cancer progression, as it serves as a substrate for oxidative metabolism, which generates more than half of the ATP that is required by malignant cells (19). As well as an important source of energy, glutamine also acts as a biosynthetic precursor for numerous molecules that are essential for rapidly proliferating cells, including fatty acids, pyrimidines, purines, and amino acids (19). Therefore, glutaminolysis is indispensable for metabolic reprogramming in cancer cells.

Mounting evidence suggests that lipid metabolism is also rewired in rapidly proliferating cells (20). The up-regulation of fatty acid synthase (FASN) and subsequent enhanced *de novo* synthesis of fatty acids have been described as a frequent event in cancer cells (21). The elevated levels of fatty acids will act as signaling molecules, energy storage, and cell membrane components, which enable tumor cells to meet the increased demands in energy and cellular components (20). Oncogenic mutations and loss of tumor suppressor genes have been shown to contribute to alterations in glutamine and lipid metabolism (11).

### Metabolic Reprogramming and Metastatic Potential of Tumor Cells

**Figure 1:**
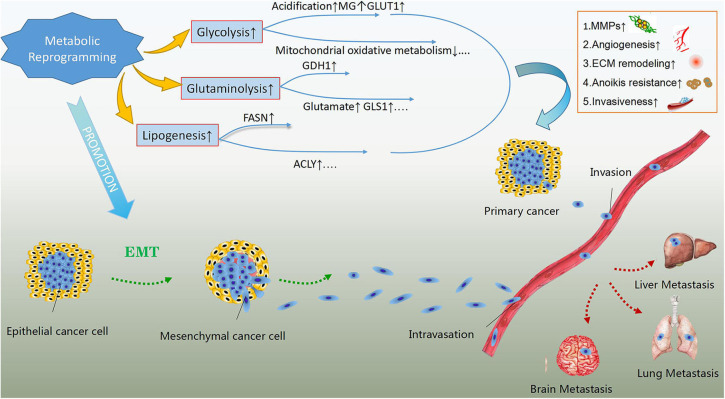
The effects of metabolic reprogramming in cancer development and metastasis. Metabolic reprogramming in cancer cells, mainly including glycolysis, glutaminolysis and lipogenesis can enhance their metastatic abilities. The products of rewired metabolism like lactate, methylglyoxal (MG) and glutamate as well as up-regulated key enzymes or concerning transporters in energy metabolism such as glutamate dehydrogenase 1(GDH1), glutaminase 1(GLS1), fatty acid synthase (FASN), ATP-citrate lyse (ACLY) and glucose transporter 1(GLUT1) are the potential regulatory mechanisms in addition to suppressed mitochondrial oxidative metabolism. These regulators can give rise to activation of matrix-metalloproteinases (MMPs), extracellular matrix (ECM) remodeling, angiogenesis, anoikis resistance, and enhanced invasiveness, which assist primary cancer cells in invading blood vessels and metastasizing to distant organs. In addition, rewired metabolism also promotes epithelial to mesenchymal transition, contributing to the metastasis of malignant cells.

Metabolic rewiring has been implicated in the enhanced ability of cancer cells to survive and proliferate, enabling them to survive under stressful conditions and resist anticancer therapies (22, 23). Importantly, increasing evidence suggests a role for metabolic reprogramming in enhancing the metastatic ability and development of cancer cells (Figure 1). Promoting the epithelial to mesenchymal transition (EMT) and subsequent detachment of cancer cells from the extracellular matrix early during metastasis are vital potential mechanisms (24). Lactate accumulation and extracellular matrix acidification due to the enhanced glycolytic rate can promote EMT by regulating the expression of EMT-related proteins (25). Moreover, extracellular acidosis can activate matrix-metalloproteinases (MMPs), which play a crucial role in the degradation of extracellular matrix and subsequent invasion of cancer cells into the vascular wall (26). The increase in the levels of the glycolysis by-product methylglyoxal, as well as the overexpression of glucose transporter 1 (GLUT1) and hexokinase 2 (HK2), have also been linked to enhanced metastatic potential (27–29). Aerobic glycolysis suppresses mitochondrial oxidative metabolism and induces anoikis resistance, promoting cancer cell migration and invasion (30).

Similarly, glutaminolysis contributes to EMT and metastasis by inducing the expression of glutamate dehydrogenase 1 (GDH1) (31). The serum levels of glutamate, the end product of glutaminolysis, have been linked to enhanced invasiveness and proliferation of prostate cancer cells (32), while in breast cancer cells, glutamate has been shown to induce migration by up-regulating the expression of transmembrane matrix metalloproteinases (33). Additionally, glutaminase 1 (GLS1), a key enzyme of glutamine metabolism, has also been implicated in cancer metastasis (34). FASN can also promote tumor cell growth and metastasis by activating the AMP-activated protein kinase (AMPK)/mTOR signaling pathway (35). The overexpression of another key enzyme of *de novo* lipogenesis, ATP-citrate lyase (ACLY), has been linked to enhanced metastatic ability and resistance to radiotherapy (36).

## Potential Mechanisms Linking Metabolic Syndrome to Cancer

### Inflammation

Inflammatory immune responses protect organisms from external pathological insults, including mechanical trauma and infectious microorganisms. In contrast to acute inflammation that has protective roles, chronic inflammation has been linked to numerous diseases, including cancer (37). Conditions caused by the disruption in metabolic homeostasis, such as central obesity and hyperglycemia, have been linked to chronic inflammation (38, 39). Anti-inflammation roles have been attributed to HDL, and a decrease in HDL levels combined with hyperglycemia can have synergistic effects in the establishment of chronic inflammation (40), linking the disruption in metabolic homeostasis to cancer development and progression.

Epidemiological studies have shown that approximately 15–20% of all cancers can be attributed to chronic inflammation or chronic infections (37). Inflammatory bowel disease (IBD), chronic hepatitis, and gastritis, respectively, elevating the risk of colorectal cancer, liver cancer, and gastric cancer are most notable examples. Inflammatory response makes a significant contribution to different stages of cancer progression (Table 1). Genetic mutations or alternations are recognized to account for most carcinogenic initiation. The long-term infiltration of inflammatory immune cells and chronic secretion of inflammatory cytokines, including tumor necrosis factor-alpha (TNF-α) and interleukins (ILs), may promote mutagenesis and abnormal gene expression (41). The increased generation of ROS by immune cells like macrophages and neutrophils during inflammation may also give rise to genetic alterations in normal cells (42). Herein, persistent inflammation can potentially predispose cells to undergo malignant transformation via inducing accumulated gene mutations. Furthermore, creating a favorable niche for transformed cells to reproduce and grow also makes a big difference in cancer development. Inflammatory cells and cytokines have been shown to induce EMT and promote cancer cell invasion (43, 44), as well as enhance cancer stemness (45), augmenting metastatic property of malignant cells. STAT3 and nuclear factor kappa B (NF-κB) signaling pathway can be activated by inflammatory responses which may mediate survival-favoring signaling and induce metabolic reprogramming, driving cancer cell growth, migration, and invasion (46, 47). The crosstalk between infiltrating inflammatory cells and malignant cells and recruitment of tumor-promoting auxiliary cells like fibroblasts confirm the decisive effect that inflammation exerts in modulating tumor microenvironment (TME) (48). Additionally, the relationship between inflammation and carcinogenesis is extremely complex, as tumors or anti-cancer therapy can also trigger inflammation via several pathways, further promoting cancer progression (62).

**Table 1:** The mechanism linking MetS to cancer.

**Subjects**	**Factors**	**Results**	**Risks**	**Reference**
Hyperglycemia	Inflammation↑	Genetic mutation↑	Carcinogenesis↑	(41, 42)
Obesity		EMT↑	Metastasis↑	(43, 44)
Dyslipidemia		Cancer stemness↑	Metastasis↑; Therapy-resistance↑	(45)
		NF-κB↑; STAT3↑	Proliferation↑; Invasiveness↑	(46, 47)
		Modulating TME↑	Carcinogenesis↑	(48)
Hyperglycemia	ROS↑	Genomic instability↑	Carcinogenesis↑	(49)
Obesity		mtDNA mutation↑	Carcinogenesis↑	(50, 51)
		Metabolic reprogramming↑	Carcinogenesis↑	(52)
		Anginogenesis↑	Metastasis↑	(53)
		EMT↑; Cancer stemness↑	Metastasis↑	(54–56)
Hyperglycemia	Cell signaling pathways↑	Wnt/β- catenin signaling↑	Carcinogenesis↑; Metastasis↑	(57, 58)
Obesity		Insulin/IGF-1 signaling↑	Carcinogenesis↑; Proliferation↑	(59)
		TGF-β signaling↑	Metastasis↑	(60)
		JAK/STAT signaling↑	Proliferation↑; Metastasis↑	(61)
		MAPK signaling↑	proliferation↑; Metastasis↑	(61)

*Metabolic syndrome or its individual components can give rise to chronic inflammation, ROS, and aberrant cell signaling pathways. And all of them are able to facilitate the initiation and progression of cancer via inducing one result or another. ↑Means enhancement or up-regulation*.

### ROS and Oxidative Stress

Reactive oxygen species (ROS) are chemically reactive molecules that are produced during oxygen metabolism. Superoxide anion (${\text{O}}_{2}^{-}$), hydrogen peroxide (H_2_O_2_), and hydroxyl radical (HO∙) are the most important ROS and are primarily produced in mitochondria by nicotinamide adenine dinucleotide phosphate (NADPH) oxidases (NOXs) (63). ROS serve as singling molecules and, in normal cells, the homeostasis in ROS levels is maintained by several cellular antioxidant mechanisms, protecting from oxidative damage. Abnormal metabolism and metabolic disorders, including hyperglycemia and adipose tissue expansion, can lead to enhanced ROS generation (64, 65).

Detection of a high ROS level in many cancer cells suggests that ROS play an essential role in cancer initiation and progression (66) (Table 1). An important mechanism by which ROS can drive carcinogenesis is the induction of DNA damage and genomic instability, contributing to malignant transformation (49). Additionally, ROS have been shown to induce mutagenesis in mitochondrial DNA (mtDNA), which has been linked to the development of certain malignancies, such as colon (50) and prostate (51) cancer. Promoting metabolic shift to glycolysis and doing damage to electron transfer may serve as underlying reasons why mutations in mtDNA can lead to tumorgenesis (52). However, only when cancer cells own the abilities to adapt to ROS stress can they survive and progress. The redox adaption including activation of redox-sensitive transcription factors like NF-κB and subsequent elevated expression of ROS-scavenging enzymes is not only responsible for cell survival but for progression as well (67). Some researchers already found ROS stress can promote angiogenesis and metastasis by inducing VEGF-A secretion and subsequent endothelial cell migration and proliferation (53). Additionally, the Wnt/β-catenin signaling pathway, which is known to drive EMT, cancer stem cell (CSC) development, and chemotherapy resistance, can be activated by ROS (54–56). Intriguingly, oncogene activation can also result in elevated ROS levels, thereby promoting cancer progression (68).

### Cell Signaling Pathways Associated With Cancer

Mutagenic signaling pathways like Mitogen-activated protein kinase (MAPK), Wnt, TGF-β, and JAK/STAT have been well-demonstrated to be critical for cancer initiation and progression (Table 1). Accumulating evidence suggests that metabolic disorders are involved in the dysregulation of these and other oncogenic pathways. In accordance with previous studies demonstrating the role of hyperglycemia in carcinogenesis, a recent study has shown that hyperglycemia can induce the Wnt/β-catenin signaling pathway, promoting cancer cell survival and the progression of hyperglycemia-related cancer (57). Obesity has also been reported to activate the Wnt/β-catenin signaling pathway in a TNF-α-dependent way (58). Moreover, aberrant insulin/insulin-like growth factor-1 (IGF-1) signaling, which has a long-standing role in cancer, is activated by hyperglycemia or obesity and is believed to be an essential mediator of the oncogenic effects of these metabolic disorders (59). Hyperinsulinemia and insulin resistance augment the activation of the MAPK pathway, promoting cancer cell proliferative survival (69). Moreover, insulin has been shown to enhance TGF-β signaling (60). In an animal model, the AMPK/mTORC1/Nox4 signaling axis was highlighted as a critical molecular mechanism linking hyperglycemia to colorectal cancer (CRC) (70). The expansion of adipocytes and subsequent disruption of adipokine signaling homeostasis has also been suggested to be an important mechanism driving the progression of cancers associated with metabolic disorders. The enhanced secretion of leptin has been linked to cancer cell growth and migration by inducing the activation of STAT3, MAPK, JAK/STAT signaling pathways (61). On the other hand, adiponectin counteracting the carcinogenic effects of leptin by interfering with the activation of oncogenic signaling pathways is remarkably reduced in obese individuals (71). In summary, the aberrant activation of oncogenic signaling pathways observed in patients with metabolic disorders represents a link between metabolic syndrome and cancer.

## Therapeutic Approaches to Target Metabolic Disorders

### Metformin

Several epidemiological studies have suggested the potential anti-cancer effects of the antidiabetic agent metformin, especially in colorectal cancer (72) and endometrial cancer (73). The mechanisms underlying the anti-cancer effects of metformin have been extensively investigated (74). Metformin administration can reduce the levels of glucose in the blood, potentially contributing to its anti-cancer effects. In a study in hepatocellular carcinoma, metformin has been demonstrated to reduce the glycolytic flux by inhibiting phosphofructokinase-1 (PFK-1), suppressing the proliferation of cancer cells (75). Additionally, metformin suppresses the expression of HIF-1α and elevation of PDH levels under hypoxic conditions, interfering with aerobic glycolysis in cancer cells (76). A study by Kitson et al. showed that metformin was capable of interfering with the function of cancer stem cells and repressing the expression of CSC-related genes (77); the inhibitory effect of metformin on CSCs is likely to be dependent on AMPK-mTOR and glutamine metabolism (78). Furthermore, a recent study suggested that the vascular effects of metformin are potential mechanisms by which metformin suppresses metastasis and sensitizes to chemotherapy (79). Metformin has also been shown to suppress cancer cell growth, as well as induce apoptosis and autophagy (80). Despite mounting evidence suggesting the potential clinical benefit of metformin for cancer prevention and treatment, Farmer et al. pointed out that different types of bias existed in many observational clinical studies on metformin and cancer (81). Notably, latest findings show that when the studies are better designed, the protective effect of metformin is attenuated (82). Accordingly, the exact efficacy of metformin in cancer and clinical application need further exploration.

### Statins

Statins are lipid-lowering agents commonly prescribed in patients with hyperlipidemia. Numerous epidemiological studies have suggested that statins may be useful in lowering the risk of cancer development. A prospective cohort study has demonstrated that statins can improve cancer patient survival and decrease the rate of cancer-related mortality in postmenopausal women (83). Patients with colorectal cancer (84) and liver cancer (85) may benefit from statins according to clinical or preclinical studies. The mechanisms underlying the anti-tumor effects of statins remain elusive. Statins are known to interfere with cholesterol synthesis by inhibiting 3-hydroxy-3methylglutary-coenzyme A(HMG-CoA) reductase. Interfering with cellular lipid biogenesis, mitochondrial metabolism, and ROS generation have been suggested as potential mechanisms by which statins may suppress cancer initiation and progression (86, 87). A study by Sadaria et al. has shown that intracellular adhesion molecule-1 (ICAM-1), an essential mediator of cancer cell metastasis, was suppressed by statins (88). Statins could also inhibit the transcription of MACC1 in colon cancer cells, suppressing cancer cell growth and metastasis (89). Importantly, statins have also been reported to have radiosensitizing effects in cancer cells (90). Autophagy evasion has been described as a hallmark of cancer, and statins have recently been shown to exert anti-cancer effects by inducing autophagy (91). A study in prostate cancer suggested that the combination of metformin and statins has synergistic effects in inducing apoptosis in chemotherapy-resistant cancer cells (92). Nevertheless, some meta-analyses demonstrated that more evidence is needed which supports the preventing role stains play in cancer (93, 94). Risk of bias must be attached more significance and be avoided in observational studies before evaluating links between statins and cancer (95). Taking all aforementioned findings into consideration, while statins seem to emerge as potential anti-cancer agents, especially for cancer patients with obesity or hypercholesterolemia, the application of statins to cancer needs further investigation.

### Lifestyle Interventions

Extensive multicenter cohort studies have demonstrated that an unhealthy lifestyle is a strong risk factor for all-cause mortality (96), including death related to MetS and MetS-associated cancer. Therefore, adopting a healthy lifestyle has been proposed as a promising approach to prevent MetS and MetS-associated malignancies. Exercise is strongly recommended, not only for reducing the risk of obesity and other MetS components but also for lowering the risk of cancer development and cancer recurrence, as well as improving the quality of life of cancer patients (97). Additionally, exercise can improve insulin resistance and modulate chronic inflammation in obese individuals (98). Undoubtedly, regular exercise is extremely important in preventing MetS and tumor development (99). Keeping a disturbed circadian rhythms at a distance is also vigorously proposed due to its intimate relation with cancer and metabolism (100). Moreover, eating too fast has been associated with hypertriglyceridemia and other MetS components (101). According to the American Dietary Guidelines, its recommended food intake patterns, moderate alcohol consumption, and a diet high in vegetables, fruits, and whole grains and low in sugar or fat, can provide a benefit to numerous chronic diseases (102). Notably, a strong link between dietary patterns and microbiota composition has been described, suggesting that a healthy diet can promote gut microbiota diversity (103), reducing the risk of MetS and cancer. In summary, increasing evidence indicates that adopting a healthy lifestyle is a potent, easy, and low economic burden mechanism for preventing MetS and cancer (104, 105).

## Discussion

As indicated by numerous epidemiological studies, along with changes in the pace and patterns of modern lifestyles, an increasing number of people are suffering from metabolic syndrome and cancer. A positive association between metabolic syndrome and cancer has been established (8). Metabolic disorders are widely accepted to be involved in the development and progression of several types of human cancers. Increased ROS production, chronic inflammation, and aberrant activation of oncogenic signaling pathways represent important links between metabolic disorders and cancer (41, 106). However, additional underlying mechanisms potentially exist, which are yet to be elucidated. For example, elevated estrogen levels in adipose tissue, as well as the increased synthesis of extracellular matrix proteins and hyaluronan (HA) in hyperglycemia, are believed to be implicated in cancer development and progression (107, 108). Cancer is strongly linked not only to alterations in metabolism at the organismal level but also at the cellular level. Cancer cells undergo metabolic reprogramming, which facilitates their malignant properties, including rapid growth, migration, and invasion (109). Given the essential role of metabolic rewiring in tumor development, drugs interfering with glycolysis, glutaminolysis, or lipogenesis, may provide a clinical benefit in cancer patients (110). Furthermore, considering the strong links between the disruption of organismal metabolic homeostasis and malignancy, therapeutic interventions already in use for patients with metabolic disorders, including metformin and statins, may serve as promising strategies to inhibit cancer development and progression. It has also become evident that a healthy lifestyle can prevent not only the development of MetS but also the development of cancer (104, 105).

In conclusion, metabolic syndrome has been established as an important risk factor for cancer development; hence, cancer patients or individuals who are at high risk of cancer development are likely to benefit from the prevention and treatment of metabolic diseases. Further efforts are required for the development of therapeutic interventions that target both aberrant metabolism and cancer.

## Author Contributions

YY and LG wrote the review, were responsible for figure and legend and final editing. JY contributes to defining the topic, analyzing article and preparing for submission. All the authors have approved for the final version of manuscript.

## Conflict of Interest

The authors declare that the research was conducted in the absence of any commercial or financial relationships that could be construed as a potential conflict of interest.

## Abbreviations

MetS: metabolic syndrome
ROS: reactive oxygen species
ATP: adenosine triphosphate
TRIM59: tripartite motif-containing59
PI3K: phosphatidylinositol 3-kinase
mtDNA: mitochondrial genome
TAM: tumor-associated macrophages
MG: methylglyoxal
GLUT1: glucose-transporter1
HK2: hexokinase 2
GLS1: glutaminase 1
MMPs: matrix-metalloproteinases
ECM: extracellular matrix
EMT: epithelial to mesenchymal transition
GDH1: glutamate dehydrogenase1
FASN: fattyacid synthase
ACLY: ATP-citrate lyase
TNF-α: tumor necrosis factor-alpha
IL: interleukin
NF-κB: nuclear factor kappa B
TME: tumor microenvironment
NADPH: nicotinamide adenine dinucleotide phosphate
CSC: cancer stem cell
MAPK: Mitogen-activated protein kinase
CRC: colorectal cancer
PFK-1: phosphofructokinase-1
HMG-CoA: 3-hydroxy-3methylglutary-coenzyme A
ICAM-1: intracellular adhesion molecule-1
HA: hyaluronan.
